# Metabolomic Analysis of Plasma From Patients With Acute Mountain Sickness Using Chromatography–Mass Spectrometry

**DOI:** 10.1097/MD.0000000000001988

**Published:** 2015-11-13

**Authors:** Guoyan Zhu, Changlin Yin, Zhu Tian, Tinggang Wang, Wei Sun, Qiang Xiang, Guoning Guo

**Affiliations:** From the Department of Health Management, Xinqiao Hospital (GZ); and Department of Emergency, Southwest Hospital, Third Military Medical University, Chongqing, China (CY, ZT, TW, SW, QX, GG).

## Abstract

Although acute mountain sickness (AMS) has long been recognized, little is known about this condition to date. The current study was conducted to explore changes in the metabolomic profiles of AMS patients and to further assess the potential of using these changes for the diagnosis of AMS.

Plasma samples from 12 patients with AMS and 12 individuals without AMS were collected and used for further bioinformatics analysis by gas chromatography–mass spectrometry (GC–MS). The following analytical methods were used: gas chromatography–mass spectrometry data preprocessing, principal components analysis, partial least squares-discriminant analysis, model validation, orthogonal partial least squares-discriminant analysis, and the screening and identification of differences in metabolites.

The results revealed a signiﬁcant difference between the subjects with AMS and those in the control group. Compared with plasma from the controls, plasma from the AMS patients contained signiﬁcantly increased hypoxanthine, cysteinylglycine, D-arabitol, L-allothreonine, 2-ketobutyric acid, and succinate semialdehyde.

The identification of metabolomic changes may be useful for the diagnosis of AMS in the future and may lay the foundation for further study of AMS pathogenesis.

## INTRODUCTION

Acute mountain sickness (AMS) is the most common high-altitude illness; it develops shortly after sudden ascent to altitudes higher than 2500 m without acclimation.^1^ Acute mountain sickness is exacerbated by hypoxic, cold environmental conditions, and fatigue. A predominant clinical characteristic of AMS is headache, which may be accompanied by nausea, vomiting, anorexia, dizziness, lethargy, fatigue, or sleep disturbance. The etiology and pathogenesis of AMS, however, are not entirely clear, and clinicians still cannot effectively diagnose AMS. Acclimation plays an important role in the occurrence of AMS. In addition to acclimation, many studies have shown that respiration, circulation, and cerebral blood flow, among other factors, changed during the occurrence of AMS. Increases in ventilation, heart function, and erythrocyte number occur to offset the inadequate oxygen level, and changes in the sympathetic nervous system and the liquid balance of the body occur for adaption to hypobaric hypoxia.

Metabolomics is an emerging discipline of systems biology that has unique advantages in the diagnosis and treatment of diseases, the determination of drug efficacies and toxicities, and the evaluation of gene functions. Through high-throughput, high-resolution, and high-sensitivity modern instrumental analysis methods such as gas chromatography–mass spectrometry (GC–MS) and nuclear magnetic resonance spectrometry combined with pattern recognition technology for assessing chemical information to analyze differences in the metabolic fingerprints of physiological and pathologic states and to identify the corresponding biomarker group. Metabolomics can reveal the functional state of the organism in a particular environment.

This study using GC–MS analysis showed that metabolic proﬁles could be used to assess biologic responses to AMS and to screen for AMS biomarkers.

## MATERIALS AND METHODS

### Subjects

The study protocol was approved by the Ethics Committee of Third Military Medical University; all subjects were well informed of the study and provided their written informed consent. Twenty-four volunteers who ascended together from the plain to Lhasa, Tibet (3658 m), by train were recruited for this clinical study. Acute mountain sickness was diagnosed by the Lake Louise self-assessment scoring system of symptoms, including headache, gastrointestinal symptoms, fatigue/weakness, dizziness/lightheadedness, and difficulty sleeping. According to the standard criteria, 12 AMS patients whose total symptom scores were above 3, and 12 individuals without AMS were selected. Both groups included healthy Han Chinese men aged between 20 and 30 years. Blood samples were collected before the patients accepted treatment.

### Plasma Sample Preparation

Taking Ethylene Diamine Tetraacetic Acid as an anticoagulant, fasting venous blood from all the above-mentioned individuals was obtained. Immediately centrifugation (3500 × *g*, 10 minutes) was performed for plasma separation. The harvested plasma samples were stored at −80 °C and transported to Shanghai for further experiments. In general, serum samples (100 μL) were combined with 0.4 mL extraction buffer and 50 μL L-2-chlorophenylalanine (0.1 mg/mL stock in dH_2_O) as an internal standard, and the samples were vortexed for 10 seconds and centrifuged for 10 minutes. Then, 0.4 mL of supernatant was transferred into a fresh 2 mL GC–MS glass vial and centrifuged at 12,000 rpm at 4 °C in preparation for testing. Gas chromatography–mass spectrometry analysis was performed once each sample aliquot was mixed.

### Gas Chromatography–Mass Spectrometry Detection

An Agilent 7890 gas chromatograph system coupled with a Pegasus high throughput time-of-flight (TOF) mass spectrometer was used to perform GC/TOF MS analysis. A 1 μL aliquot of analyte was injected in splitless mode. Using helium as the carrier gas, the initial temperature was maintained at 50 °C for 1 minute, raised to 330 °C at a rate of 10 °C min^−1^, and then maintained at 330 °C for 5 minutes. The mass spectrometry data were acquired under such circumstances: under full-scan mode; energy of electron impact mode: −70 eV; *m/z* range: 85 to 600; rate: 20 spectra per second; solvent delay: 366 seconds. ChromaTOF 4.3X software (LECO Corporation) and the LECO/Fiehn Rtx5 database were used for raw peak extraction, baseline data filtering and calibration, peak alignment, deconvolution analysis, peak identification, and peak area integration. The retention time index method was used in the peak identification; the retention time index tolerance was 5000.

### Principal Components Analysis and Partial Least Squares Discriminant Analysis

SIMCA-P + software (V13.0, Umetrics AB, Umea, Sweden) was used for the multivariate analysis after data normalization and pattern recognition, and principal components analysis (PCA) was performed using the Ctr-formatted (mean-centered scaling) data scale conversion method.^2^ Automatic data modeling and analysis were conducted, and partial least squares-discriminant analysis (PLS-DA) was used to identify the correlation between the data (*X* variable) and other factors (*Y* variables, grouping information) using the same software. Partial least squares-discriminant analysis using the Ctr-formatted data scale conversion method was then performed to evaluate the models for the first and second principal components (PCs). The quality of PLS-DA on the model was tested using leave-one-out cross-validation. After cross-validation, R^2^X (explainable index) and *Q*^2^ (predictable index) values were obtained to judge the validity of the model. The validity of the model was further tested by arranging several randomized experiments (n = 200) to obtain corresponding values of different random *Q*^2^ values by changing the order of the categorical variables.^3^

### Orthogonal Partial Least Squares-Discriminant Analysis

Orthogonal partial least squares-discriminant analysis was used to adjust the processing for the PLS-DA model according to SIMCA-P + software. Orthogonal partial least squares-discriminant analysis was applied to maximize and highlight the internal differences between the AMS and control groups. Orthogonal partial least squares-discriminant analysis data were formatted using the Ctr-formatted (mean-centered scaling) data scale conversion method to analyze the models for the first and second PCs.^4^

### Screening and Identification of Differences in Metabolites

Orthogonal partial least squares-discriminant analysis was performed by filtering irrelevant quadrature signals; thus, the observed differences in metabolites were more reliable. This project used the first PC of variable importance in the projection (VIP) values. (threshold >1) of the OPLS-DA model combined with the *P* value determined by Student's *t*-test (threshold 0.05) to identify differentially expressed metabolites.

## RESULTS

**FIGURE 1:**
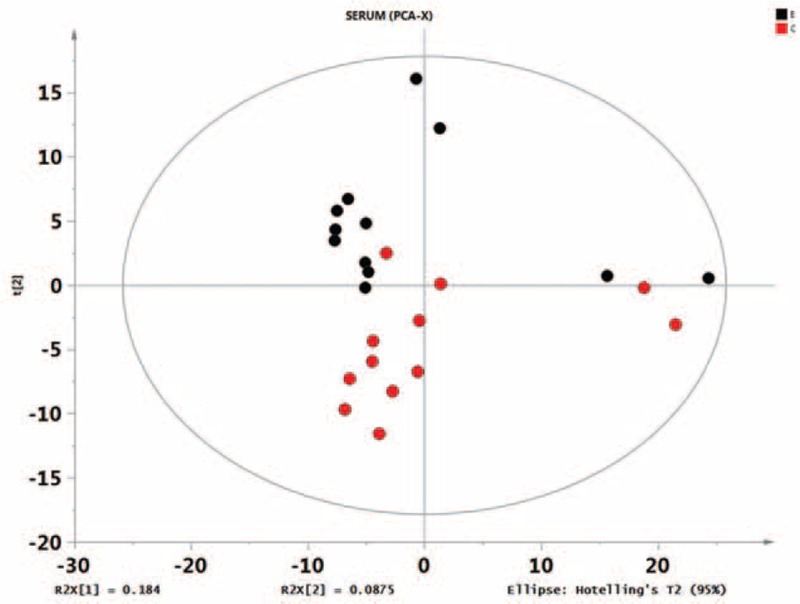
Score plot of the principal components analysis model obtained from E and C. The principal components analysis score plot of the raw data samples is an overall presentation of the distribution. As can be observed from the scores, each group of samples can be substantially differentiated; some groups may be indistinguishable from others by subsequent discriminant analysis. Overall, these data are worthy of further study.

**FIGURE 2:**
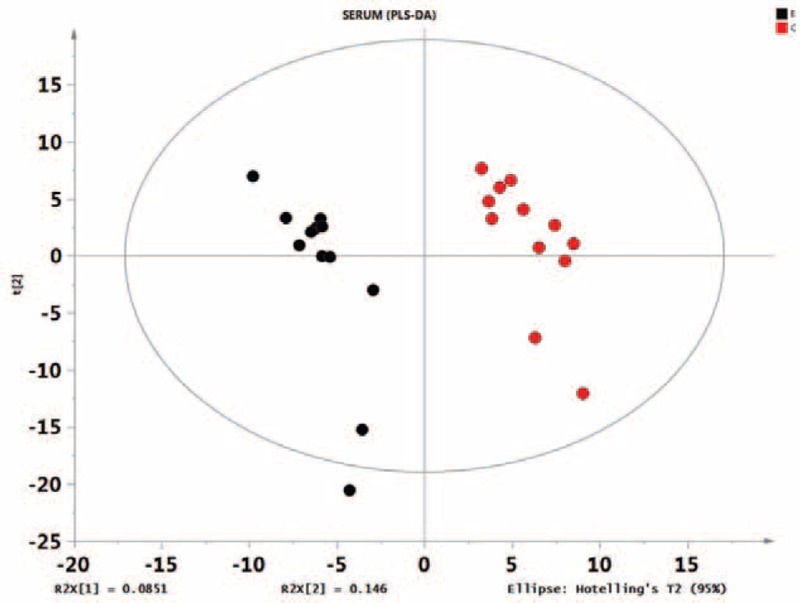
Score plot of the PLS-DA model obtained from E and C. The PLS-DA scores are shown in Figure 2 (horizontal axis shows the first principal component scores, with *t* [1] represented; vertical axis shows the second principal component scores, with *t* [2] represented). The values of R^2^X (interpretable variable model) and R^2^Y (which is explained by models of supervision) are near 1, which shows that the PLS-DA model can well explain the differences between the 2 groups. The permutation test intercept (*R*^2^ = 0.889, *Q*^2^ = 0.0694) could very well reflect the validity of the model, indicating that the basic data are not overfit. PLS-DA, partial least squares-discriminant analysis.

**FIGURE 3:**
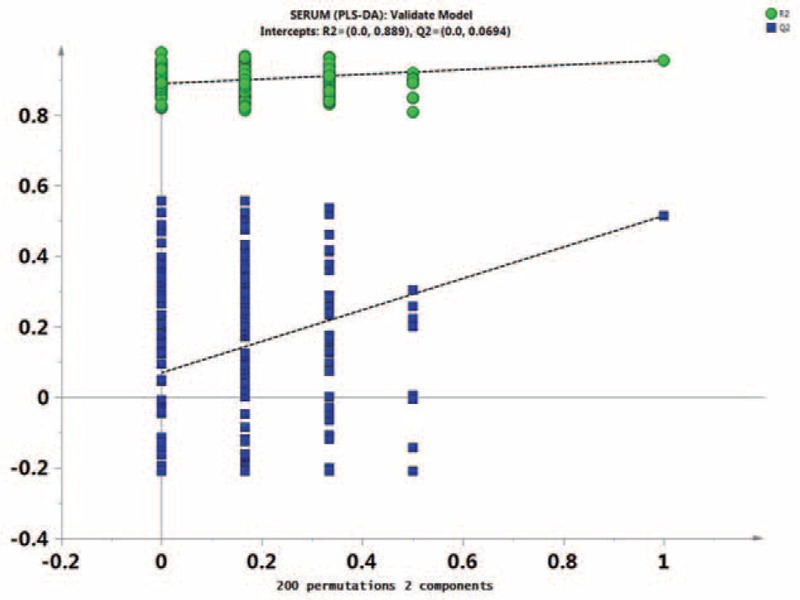
Permutation test of the partial least squares-discriminant analysis model obtained from E and C. Two hundred permutations were performed, and the resulting *R*^2^ and *Q*^2^ values were plotted. Green triangle: *R*^2^; blue square: *Q*^2^. The green line represents the regression line for *R*^2^, and the blue line represents that for *Q*^2^.

**FIGURE 4:**
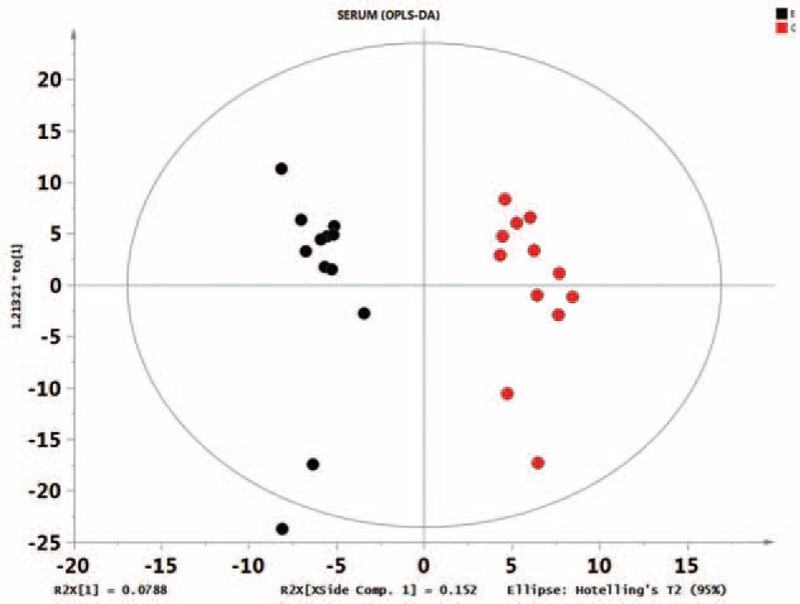
Score plot of the orthogonal partial least squares-discriminant analysis model obtained from E and C. A loading plot, which showed the contribution of the variables to the difference between 2 groups, was constructed.

First, missing raw data values were replaced by half of the minimum value; 429 peaks were detected, and 419 metabolites remained following the application of an interquartile range denoising method. In addition, the internal standard normalization method was used in this data analysis. The resulting three-dimensional data, including the peak number, sample name, and normalized peak area, were entered into the SIMCA-P 13.0 software package (Umetrics, Umea, Sweden) for PCA, PLS-DA, and OPLS-DA. Principal components analysis showed the distribution of the original data (Figure 1). A supervised PLS-DA was applied to obtain a higher level of group separation and a better understanding of the variables responsible for classification (Figure 2). The parameters for classification from the software were R^2^Y = 0.955 and Q^2^Y = 0.515 (Figure 3), which were stable and good in terms of fitness and prediction. Robustness and the predictive ability of our model were estimated by 10-fold cross-validation, and a permutation test was conducted to further validate the model. The *R*^2^ and *Q*^2^ intercept values were 0.889 and 0.0694, respectively, after 200 permutations. The low values of the *Q*^2^ intercept indicate the robustness of the models, indicating a low risk of overfitting and high reliability. A loading plot was constructed based on OPLS-DA (Figure 4), revealing contribution of variables to differences between 2 groups. The plot also showed the important variables, which were situated far from the origin; however, the loading plot is complex because of the large number of variables. First, VIP PC was obtained to refine this analysis. Changed metabolites were first selected when the VIP values exceeding 1.0. In step 2, Student's *t*-test (*P* > 0.05) was then assessed to the remaining variables. Variables were discarded between 2 comparison groups. In addition, commercial databases, including the Kyoto Encyclopedia of Genes and Genomes (KEGG; http://www.genome.jp/kegg/) and National Institutes of Standards and Technology (NIST; http://www.nist.gov/index.html) databases, were used to search for metabolite pathways. In the AMS group, we found 19 altered metabolites, 6 of which were at significantly increased levels compared with those in the control group, hypoxanthine, cysteinylglycine, D-arabitol, L-allothreonine, 2-ketobutyric acid, and succinate semialdehyde, with the remaining 13 at significantly decreased levels compared with the control group: 2-monopalmitin, 1-monopalmitin, galactose, 3-hydroxybutyric acid, alizarin, L-homoserine, maleamate, tryptophan, glycine, asparagine, biuret, caffeic acid, and 2-amino-2-norbornane carboxylic acid 4 (Table 1).

**TABLE 1:**
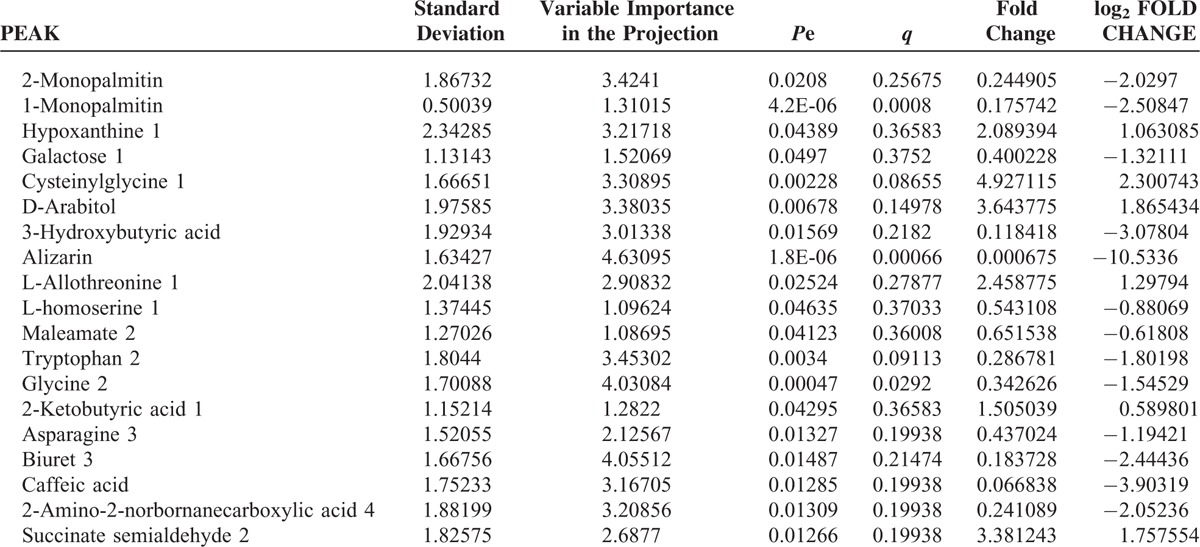
Screening and Identification of Differences in Metabolites

## DISCUSSION

Acute mountain sickness is the most common of the high-altitude illnesses and is mainly characterized by headache. Other symptoms include the following: nausea, vomiting, anorexia, dizziness, lethargy, fatigue, and sleep disturbance. Acute mountain sickness is likely to occur without acclimation.^5,6^ Many factors in the highland environment have adverse effects on the human body, including thin, cold and dry air, increased ultraviolet radiation, and strong winds. Among these factors, hypoxia induced by thin (low oxygen) air is the greatest danger. Hypoxia causes headache, chest tightness, shortness of breath, dizziness, insomnia, nausea, vomiting, and loss of appetite. If AMS is not diagnosed and treated in a timely manner, the deadly conditions high-altitude cerebral edema and high-altitude pulmonary edema will likely occur. Thus far, the diagnosis of AMS has mainly depended on the presence of symptoms and on oxygen saturation. If prevention and treatment strategies are successfully implemented, AMS can be controlled, even in a large population. Speciﬁc biomarkers, however, would be useful for the early diagnosis of AMS.

Gas chromatography–mass spectrometry combines the separation ability of chromatography and the qualitative strengths of mass spectrometry and can be used to qualitatively analyze a multicomponent mixture in a short period of time. Gas chromatography–mass spectrometry can detect almost all compounds and can accurately determine the molecular mass, chemical formula, and molecular structure; this method also has the advantages of high sensitivity and qualitative abilities. In this study, we analyzed the metabolic proﬁles of patients with AMS using GC–MS. The score plot results showed that the plasma metabolic profiles of the 2 groups were obviously different. This result demonstrates that the metabolic profile in the plasma of AMS patients is altered.

Regarding sugar metabolites, D-arabitol was increased but galactose was decreased in the plasma of AMS patients, suggesting that acute hypoxia altered glucose metabolism. According to metabolic profiles, glucose and lactate are the predominant energy sources in most organisms. During acute hypoxia, plasma glucose was increased, which correlated with inhibited glucose aerobic oxidation; however, glycogenolysis and gluconeogenesis were enhanced.

In addition, lipids, including 2-monopalmitin, 1-monopalmitin, and 3-hydroxybutyric acid, were obviously decreased in patients with AMS compared with those in healthy controls. The decrease in lipids may prevent energy support at high altitudes, which results in adenosine triphosphate insufficiency and is a potential contributor to AMS.

In this study, the levels of a wide variety of amino acids were altered by the pathogenesis of AMS compared with their levels in healthy individuals. In the AMS group, 3-hydroxybutyric acid, L-homoserine, maleamate, tryptophan, glycine, asparagine, caffeic acid, and 2-amino-2-norbornanecarboxylic acid were significantly decreased, whereas cysteinylglycine, L-allothreonine, 2-ketobutyric acid, and succinate semialdehyde were increased in the plasma metabolic profiles. A large number of metabolites are involved in amino acid metabolism because of its complexity. The disorder of amino acid metabolism can be caused by the dysregulation of proteolysis, oxidative catabolism, and gluconeogenesis.^7^ Cysteinylglycine, which is synthesized from methionine and serine, can decompose into taurine. Taurine is a sulfuric amino acid with strong cytoprotective antioxidant activity, which can protect cell membranes and other cell components. Our results showed that cysteinylglycine was increased in the plasma of AMS patients, suggesting that taurine levels were also elevated.^8^ Although L-allothreonine is not an essential amino acid in the human body, it plays an important role in fat and fatty acid metabolism as well as in muscle growth and contributes to immunoglobulin and antibody production. In AMS, the decreased L-allothreonine levels may reduce the immune function of AMS patients; however, the exact mechanisms must be further explored. After being oxidated, tryptophan can be converted into serotonin, which can then lead to microvascular contraction and high blood pressure, can reduce fructose absorption and can accelerate the rate of protein synthesis. Glycine is an inhibitory neurotransmitter in the central nervous system. Tryptophan and glycine are essential amino acids of the human body and are referred to as a pair of “sleeping amino acids” because they can promote sleep. We found decreased plasma tryptophan and glycine levels, which may be related to the sleep disorders found in AMS patients. L-Allothreonine is an essential amino acid that can aid in alleviating human fatigue, promote growth and development, play an important role in water holding in human cells, and protect the cell membrane. Furthermore, L-allothreonine can promote phospholipid synthesis as well as fatty acid oxidation. 2-Ketobutyric acid plays an active intermediate role in organic synthesis and biologic processes. In the current study, L-allothreonine and 2-ketobutyric acid were significantly increased in AMS plasma samples, suggesting that these 2 amino acids may play antagonistic roles against AMS. Succinate semialdehyde-2 is an enzyme that is produced by the inactivation of γ-aminobutyric acid transaminase, which plays an inhibitory role in the central nervous system. Our results showed increased succinate semialdehyde-2 levels in the plasma of AMS patients, indicating a reduction in the inhibitory effect of γ-aminobutyric acid transaminase on the central nervous system. Overexcitement of the central nervous system is characterized by nervous system disorders, high blood pressure and sleep disorders. The increased succinate semialdehyde-2 level combined with the reduced tryptophan and glycine levels in the plasma cause sleep disorders.

## CONCLUSIONS

To summarize, in AMS patients, aerobic metabolism is inhibited, whereas anaerobic glycolysis is increased. Changes in the levels of a number of related metabolites, such as plasma cysteinylglycine, D-arabitol, succinate semialdehyde, and alizarin are obvious, but the specific regulatory sites and regulatory mechanisms that are involved and the biologic significance of such factors require further study.

Despite the limitation of the small sample size, this study illustrates the useful application of metabolomics analysis, based on GC–MS of blood plasma samples, for the investigation of metabolic changes in AMS patients. Moreover, this study suggests that metabolite analysis could provide a new understanding of AMS and may be useful in the diagnosis and treatment of AMS.
